# Sensitivity of Awaji Criteria and Revised El Escorial Criteria in the Diagnosis of Amyotrophic Lateral Sclerosis (ALS) at First Visit in a Tunisian Cohort

**DOI:** 10.1155/2021/8841281

**Published:** 2021-01-22

**Authors:** Bademain Jean Fabrice Ido, Imen Kacem, Mahamadi Ouedraogo, Amina Nasri, Saloua Mrabet, Amina Gargouri, Mouna Ben Djebara, Bawindsongré Jean Kabore, Riadh Gouider

**Affiliations:** ^1^Department of Neurology, University Hospital Yalgado Ouedraogo, Ouagadougou, Burkina Faso; ^2^Department of Neurology, UR12SP21, Razi Hospital, Manouba, Tunisia

## Abstract

**Background:**

Amyotrophic lateral sclerosis (ALS) is a fatal disease whose diagnosis and early management can improve survival. The most used diagnostic criteria are the revised El Escorial criteria (rEEC) and Awaji criteria (AC). The comparison of their sensitivities showed contradictory results. Our study aimed to compare the sensitivities of these two criteria in the diagnosis of definite ALS, at first visit, in a Tunisian hospital cohort.

**Materials and Methods:**

This was a retrospective study including 173 patients diagnosed with ALS at the Department of Neurology of the Razi Hospital between January 2003 and April 2018.After studying the clinical features of the disease in our study population,each patient was categorized according to the rEEC and AC based on data collected in his medical record during his first visit to our department. Then, we compared the sensitivities of these two criteria in the diagnosis of definite ALS.

**Results:**

Our Tunisian cohort was characterized by a slower disease progression. The sensitivity of the AC (69.4%) was significantly higher than that of the rEEC (40.5%) (*p* < 0.001). When the clinical signs evolved for less than 6 months, the sensitivities were 61% for AC and 12% for rEEC (*p* < 0.001). After 24 months of disease progression, the sensitivities were 78.2% for AC and 69.1% for rEEC (*p* = 0.063). It was impossible to categorize seventeen patients by the two criteria.

**Conclusion:**

Our study demonstrated that patients in AC are more sensitive than rEEC in the early diagnosis of ALS in our Tunisian cohort. However, this superiority is gradually reduced during the evolution of the disease.

## 1. Introduction

Amyotrophic lateral sclerosis (ALS) is a neurodegenerative disease defined by the association of upper motor neuron (UMN) and lower motor neuron (LMN) signs in the bulbar and spinal territories, which typically leads to death with a median survival of 36 months [1].

Early treatment can improve survival, thanks to a few drugs including riluzole and more recently edaravone, which has been approved since 2017 [2].

Sometimes the early diagnosis of ALS is still difficult because there is no specific diagnostic test or biomarker for the disease and because of the heterogeneity of the phenotypes.

This difficulty led to the organization of the El Escorial Consensus conference in 1991 and 7 years later that of Airlie House, which, respectively, proposed the El Escorial criteria [3] and revised El Escorial criteria (rEEC) [4] to harmonize protocols and therapeutic trials and provide as early as possible the proper care to patients.

However, these criteria have been considered insufficient by several authors [5, 6] because of the low diagnostic sensitivity and the lack of importance to electromyography (EMG) signs, particularly the fasciculation potentials recorded during EMG examination.

Thus, the Awaji criteria (AC) were proposed in 2008 [7], which accepted the existence of LMN involvement as well based on the presence of clinical signs as on the presence of EMG signs including fasciculation potential.

Since then, many studies have compared the diagnostic sensitivities of AC and rEEC, with contradictory results [8–13], which could be related to the differences between the study populations and methodological limitations.

The aim of the present study was to compare the sensitivities of AC with rEEC in the diagnosis of definite ALS, at first visit, in a Tunisian hospital cohort.

## 2. Patients and Methods

It was a retrospective study carried out at the neurology department of the Razi Hospital of La Manouba in Tunis (Tunisia). We included 173 patients admitted between January 2003 and April 2018 with anamnestic, clinical, and electrophysiological arguments in favor of ALS. We reviewed the clinical, electrophysiological, biology (metabolic, immunological, and paraneoplastic), and imagery (cerebral and/or medullary magnetic resonance imaging (MRI)) data, to exclude potential ALS mimic disorders. The demographic, clinical, and first EMG data of those patients have been collected from their medical record. The juvenile form of ALS corresponds to cases where the age of onset was <25 years. The classic form of ALS corresponds to all the other cases (age of onset ≥25 years) [14–16].

All the EMG data were made by the same team from 2003 to 2018 according to the same protocol.

Patients whose EMG techniques were not comparable to the recommendations of the AC were excluded.

Each patient was categorized according to the AC and the rEEC based on clinical and electrophysiological data collected in his medical record during his first visit to our department.

For the calculation of diagnostic sensitivities, each of the diagnostic criteria was regarded as positive if the patient was categorized “definite ALS” the first time he was admitted at the neurology department of Hospital Razi of La Manouba.

For all other patients categorized “probable ALS,” “probable laboratory-supported ALS,” or “possible ALS,” the diagnostic criteria were considered negative.

The reference standard we use to confirm the diagnosis was disease progression follow-up in our department as determined by history and examination.

Then, we compared the diagnostic sensitivity of these two diagnostic criteria.

The statistical analyses were performed using SPSS 22.0. McNemar test was used to determine the statistical significance of the sensitivity's differences. *p* < 0.05 was considered as statistically significant.

## 3. Results

### 3.1. Epidemiological Characteristics of the Study Population

In our cohort of 173 patients, there was a male predominance with 108 men (62.4%) and 65 women (37.6%). The sex ratio (male/female) was 1.6.

The mean age at onset was 54.1 (±14.6) years. The mean age of patients at the first visit was 56.9 (±13.8) years.

The average time to diagnosis of the disease was 33.7 months (±43.9). The median survival in our cohort was 56 months.

The classic form was found in 163 patients (94.2%), while 10 patients (5.8%) presented the juvenile form. The spinal onset patients were predominant (81.5%), and the bulbar onset form represented 16.8%.

### 3.2. Comparison between AC and rEEC

#### 3.2.1. For the Entire Cohort of ALS Patients

**Figure 1:**
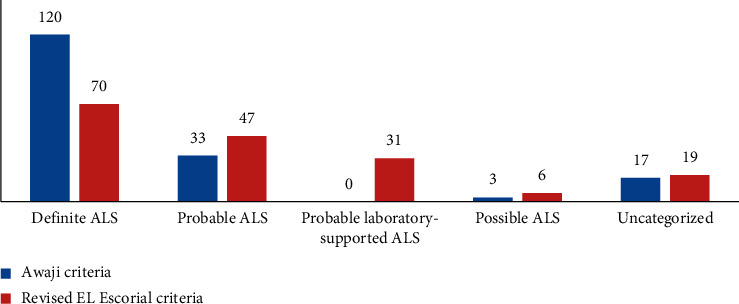
Diagnostic categories for the entire cohort of ALS patients, according to the AC and rEEC.

In our cohort of 173 patients, 120 patients were classified as “definite ALS” according to the AC, and 70 patients according to the rEEC. The sensitivity was 69.4% for the AC and 40.5% for rEEC. The difference was significant (*p* < 0.001) (Figure 1).

#### 3.2.2. According to the Onset Form

In the spinal onset patients, the sensitivity was 70.2% for the AC and 39.8% for the rEEC, while in the bulbar onset patients, the sensitivity was 65.6% for the AC and 42.1% for the rEEC. The difference was significant (*p* < 0.001).

#### 3.2.3. According to the Duration of Evolution of the Disease at the First Visit

**Figure 2:**
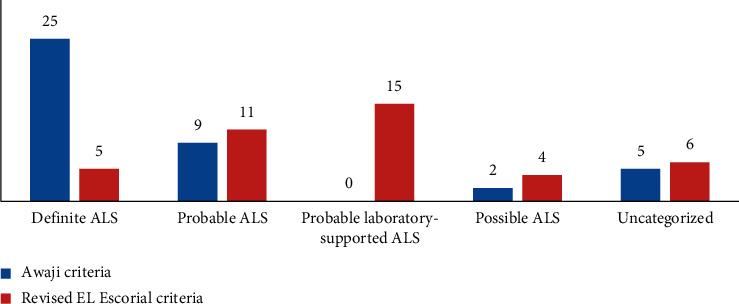
Diagnostic categories for ALS patients with a duration of evolution of the disease from 0 to 6 months, according to the AC and rEEC.

*(1) For those who had a duration of evolution of the disease from 0 to 6 months*: in a cohort of 41 patients, 25 patients were classified as “definite ALS” according to the AC and 5 patients according to rEEC (Figure 2). The sensitivity was 61% for the AC and 12.2% for rEEC. The difference was significant (*p* < 0.001).

**Figure 3:**
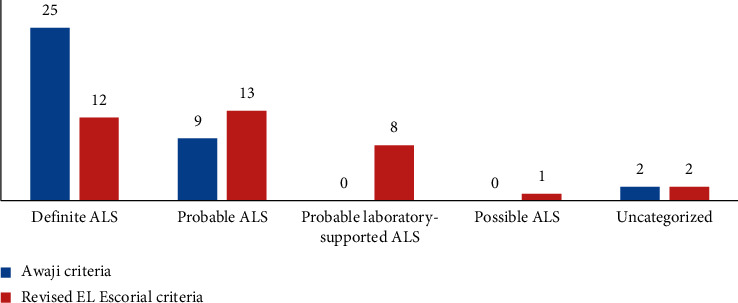
Diagnostic categories for ALS patients with a duration of evolution of the disease from 7 to 12 months, according to the AC and rEEC.

*(2) For those who had a duration of evolution of the disease from 7 to 12 months:* out of a total of 36 patients, 25 patients were classified as “definite ALS” according to the AC and 12 patients according to the rEEC (Figure 3). The sensitivity was 69.4% for the AC and 33.3% for rEEC. The difference was significant (*p* < 0.001).

**Figure 4:**
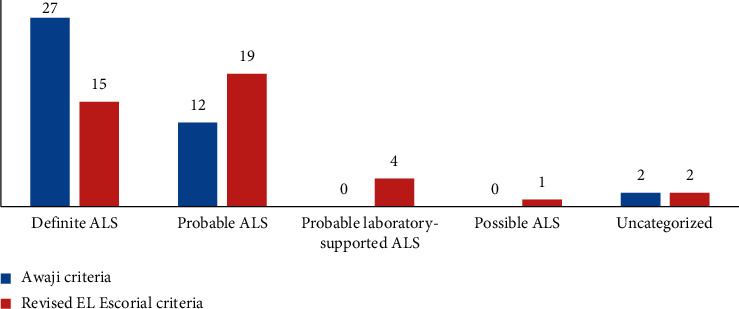
Diagnostic categories of ALS patients with a duration of evolution of the disease from 12 to 24 months, according to the AC and rEEC.

*(3) For those who had a duration of evolution of the disease from 13 to 24 months*: out of a total of 41 patients, 27 patients were classified as “definite ALS” according to the AC and 15 patients according to the rEEC (Figure 4). The sensitivity was 65.9% for the AC and 36.6% for rEEC. The difference was significant (*p* < 0.001).

**Figure 5:**
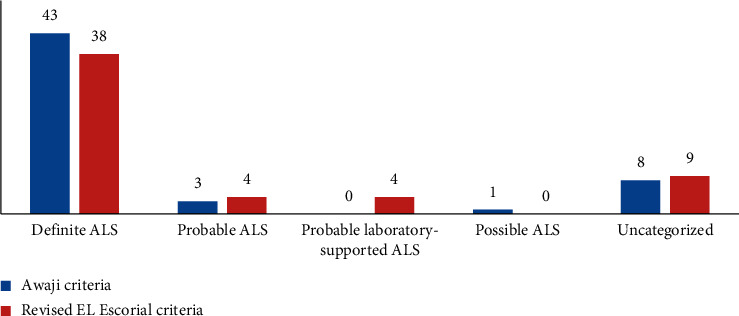
Diagnostic categories of ALS patients with a duration of evolution of the disease exceeding 24 months, according to the AC and rEEC.

*(4) For those who had a duration of evolution of the disease exceeding 24 months*: out of a total of 55 patients, 43 patients were classified as “definite ALS” according to the AC and 38 patients according to the criteria of rEEC (Figure 5). The sensitivity was 78.2% for the AC and 69.1% for rEEC. The difference was not significant (*p* = 0.063).

#### 3.2.4. Patients Who Were Impossible to Categorize

In our cohort, it was impossible to categorize 17 patients by the two diagnostic criteria at their admission. Of these patients, 16 had no evidence of UMN involvement and 1 patient had no evidence of LMN involvement. The distribution according to the duration of evolution shows that these patients were more numerous in the groups of those who consulted earlier (5 patients) and later (8 patients).

## 4. Discussion

In this study, 173 cases of ALS were collected between 2003 and April 2018. It is one of the African largest monocentric cohorts found in the literature [11, 17].

The type of study could be biased since it seems difficult to demonstrate that all the EMG data completed during our study period met the recommendations of the AC. However, we think that this bias can be minimized because all these EMG data were made by the same team from 2003 to 2018 according to a well-codified protocol. Patients whose EMG techniques were not comparable to the recommendations of the AC were excluded.

### 4.1. Epidemiological Characteristics of the Study Population

In our cohort, there was a male predominance. The spinal onset form predominated (81.5%) followed by the bulbar onset form (16.8%). All these results are the same as those found in the literature [18, 19]. However, the mean age of patients at the onset of the disease was 54.1 years (±14.6), while in Western series, this age varies between 60 and 65 years [20]. This difference could be explained by the fact that the African population in general is much younger than the European one, with a lower life expectancy [21]. The hypothesis of a genetic implication could also be raised.

The average time to diagnosis of the disease was 33.7 ± 43.9 months. Li et al. [12] and Noto et al. [12] found, respectively, 11 and 15 months. In addition, the median survival in our cohort was 56 months, and this is much higher than that found in the literature [18, 22, 23]. The TROPALS study [21] confirms our results to a lesser extent by showing that the patients of North Africa have a median survival from the onset of the disease which is at 37 months (95% CI 25.8 at 48.1 months), which is higher than for patients from other regions of Africa and Europe. The fact that, in our cohort, the median survival is very high could be explained first by the fact that the young age of the patients in the classic form is a factor of improvement of survival [22]. Then, the juvenile form is relatively frequent in our cohort, whereas this form would have a slower evolution [17, 23]. Finally, the hypothesis of a genetic implication could also be raised.

### 4.2. Comparison between AC and rEEC

For the entire cohort, our study confirmed the higher diagnostic sensitivity of the Awaji criteria found in most of the studies that compared the two diagnostic criteria [8, 12, 19, 24]. This is certainly what led Ludolph et al. [25] to propose in 2015 another revision of rEEC, which has not yet been adopted by most clinicians, but which could increase the sensitivity of the criteria proposed in 2000. However, other studies found no significant difference between the two criteria we compared [9, 10]. This inconsistency may be related firstly to differences in study populations; for instance, our study showed a slower evolution of the disease in the Tunisian population. Secondly, the difference in study methods may explain this discrepancy of the results because the definitions of the sensitivity of the tests vary from one study to another [26]. Additionally, when comparing the diagnostic sensitivities of the two criteria according to the duration of evolution of the disease, we note that, for the first 6 months of the disease, AC are 5 times more sensitive than rEEC. For patients, whose disease has been evolving for 12 months, this difference is less marked with sensitivity at 65.9% and 36.6%, respectively, for AC and rEEC. After 2 years of evolution of the disease, there is no longer any significant difference between the two criteria. This variation in sensitivity of the criteria according to the duration of evolution of the disease could also explain the differences in sensitivity found in the studies. Indeed, most authors did not compare the two tests according to the duration of evolution of the disease. The low sensitivity of rEEC criteria at the onset of the disease could be explained by the fact that, at this stage, the signs of LMN involvement are generally only subclinical fasciculations, which are only detectable by EMG and ultrasound [27]. Clinical signs of LMN impairment such as muscle weakness and muscular atrophy appear when at least a third of LMN have degenerated [28, 29]. This is certainly also the reason why, for those who had a longer duration of the disease, the difference between the 2 criteria was not significant. Indeed, at this stage, the degeneration of LMN is sufficiently advanced for the signs to be present both clinically and electromyographically. These results support the importance of EMG in the early diagnosis of ALS [8, 12, 30].

The sensitivity of the AC remains higher than that of the rEEC, whatever the onset form. However, the sensitivity of the AC is lower in bulbar onset patients than in spinal onset patients, while it is the opposite for the rEEC.

This can be due to the fact that, in the bulbar onset form, the clinical signs of LMN impairment (dysphonia and dysphagia) are more evident than in the spinal onset form, which is the opposite for EMG signs of LMN impairment.

Seventeen patients were impossible to categorize by the two criteria because they had no signs of impairment of UMN or LMN at the time of our examination. This shows that the two diagnostic criteria have limits. Therefore, Shefner et al. [31] have introduced in 2020 new criteria that seem easier to apply.

The distribution of the uncategorized patients according to the duration of the disease shows that they were numerous in the groups of those who consulted earlier and later. This can be explained by the slow clinical course of the disease in our patients. In addition, among these 17 uncategorized patients, 16 of them had no sign of impairment of the UMN. This may be because at least partial LMN integrity is required to ascertain the clinical signs suggestive of UMN involvement, which is not always the case in patients with ALS [32].

## 5. Conclusion

Our study showed that, for all the patients in our cohort, sensitivity of AC is higher than the rEEC in the diagnosis of definite ALS at the first visit.

However, the sensitivity of the two diagnostic criteria changes according to the duration of evolution of the disease. Indeed, the first 6 months of the disease AC are 5 times more sensitive than the rEEC, whereas after 2 years of evolution of the disease, there is no more significant difference between the 2 diagnostic criteria.

These results support the importance of EMG in the early diagnosis of this disease in the Tunisian population.

## Data Availability

The original relevant data are present within the paper.

## Abbreviations

ALS: Amyotrophic lateral sclerosis
rEEC: Revised El Escorial criteria
AC: Awaji criteria
UMN: Upper motor neuron
LMN: Lower motor neuron
EMG: Electromyography
MRI: Magnetic resonance imaging.
